# Supplementary material for: Diagnostic Associations of Processing Speed in a Transdiagnostic, Pediatric Sample
Source: Sci Rep. 2020 Jun 22;10:10114. doi: 10.1038/s41598-020-66892-z (PMC7308370; doi:10.1038/s41598-020-66892-z)
Supplement: Supplementary file 1 — Supplementary Information. [file 41598_2020_66892_MOESM1_ESM.pdf]

Associations between Processing Speed and Psychopathology in a Transdiagnostic, Pediatric Sample

Authors: [1]Eliza Kramer, [2]Bonhwang Koo, [1]Anita Restrepo, [3]Maki Koyama, [1]Rebecca Neuhaus, [3]Kenneth Pugh, [1]Charissa Andreotti, [2, 4]Michael Milham*

[1]Healthy Brain Network, Child Mind Institute, New York, New York
[2]Center for the Developing Brain, Child Mind Institute, New York, New York
[3]Haskins Laboratories, New Haven, CT
[4]Center for Biomedical Imaging and Neuromodulation, Nathan S. Kline Institute for Psychiatric Research, Orangeburg, New York

Supplemental Material

Table of Contents

Supplementary Text 3

Table S1: Variable loadings for the four subtests onto the PCA components. 4

Table S2: PC1 regressed on categorical diagnostic labels, with FSIQ included as a covariate. 5

Table S3: PC1 regressed on dimensional measures, with FSIQ included as a covariate. 6

Table S4: PC1 regressed on categorical diagnostic labels with SWAN-inattention and FSIQ included as covariates 7

Table S5: Correlations among processing speed tasks, potential confounds and dimensional symptoms . 8

Table S6: Each of the four processing speed tasks regressed separately on categorical diagnostic labels 9

Table S7: Each of the four processing speed tasks regressed separately on dimensional measures……...11

Table S8: Each of the four processing speed tasks regressed separately on categorical diagnostic labels, including SWAN-IN as a covariate 13

Table S9: Correlations between PC2 and four individual PS measures, potential confounds, and dimensional symptoms 15

Table S10: PC2 regressed on categorical diagnostic labels 16

Table S11: PC2 regressed on dimensional measures 17

Supplementary Text

S1: This table shows the variable loadings of the four subtests onto the four PCA components.

S2-4: Multiple linear regressions examined the same analyses run in the main paper on PC1, while also including FSIQ as a covariate. Though we believe results should be examined without FSIQ given the intricate relationship between intelligence and many of these variables, these tables are included to show that the major findings largely remained intact when overall intelligence was taken into account. Table S2 examines how categorical diagnosis from HBN clinicians relates with PC1. Table S3 examines which dimensional symptoms from parent and self-report questionnaires are related to PC1. Table S4 shows results from the regression analysis which examines relationships between categorical diagnoses and PC1 while controlling for inattentive symptoms.

S5-8: The four tables in this section outline the same analyses run on PC1, but with mental health symptoms and diagnoses predicting the four processing speed tasks individually. These tables assist with comparing results across the literature for publications that only use one of the PS tests. Table S5 shows the relationships between the four PS tests and variables of interest, including confounding variables and dimensional symptoms. The following three tables (S6-8) show the results of multiple linear regression models predicting the four PS tests individually, with and without FSIQ as an added covariate.

S9-11: S9-11 tables show the relationship between PC2 and pediatric mental health. PC2 explained only 19% of the variance of the four tasks and thus was not included in the main manuscript. Table S9 shows correlations between PC2, confounding variables and dimensional symptoms. Table S10 shows the linear regression examining relationships between categorical diagnosis and PC2, with and without including FSIQ as an added covariate. Finally, Table S11 shows the linear regression examining how dimensional symptoms relate with PC2, with and without FSIQ as an added covariate. Given that PC2 is not related to inattentive symptoms, further regressions specifically examining inattention were not included.

For all analyses, please refer to the following to indicate level of significance: $p < 0.05$ *, $p < 0.01$**, $p < 0.001$***

Table S1: Variable loadings for the four subtests onto the PCA components

PCA Variable Loadings				
	**PC1**	**PC2**	**PC3**	**PC4**
**WISC-Coding**	0.5336653	-0.1405348	0.6610039	-0.50845374
**WISC-SS**	0.5497116	-0.1753970	0.1380940	0.80497399
**NIH PC**	0.3905519	0.9063796	-0.1553503	-0.04256243
**WISC-SS EEG**	0.5103801	-0.3577176	-0.7210202	-0.30278723

Table S2: PC1 regressed on categorical diagnostic labels, with FSIQ included as a covariate

F statistic = 39.82, df =785, adjusted R^2 = 0.39

variable	b value	SE	p value
(Intercept)	-3.591	0.4265	1.78E-16***
ADHD-I	-0.4422	0.1011	1.39E-05***
ADHD-C	-0.2837	0.1092	0.009556**
ASD	-0.1383	0.1232	0.2621
SLD-Math	-0.3774	0.1102	0.0006513***
SLD-Reading	-0.08461	0.1105	0.4442
Anxiety	-0.01889	0.09313	0.8393
Depression	0.06272	0.1581	0.6917
Sex	0.4091	0.08994	6.26E-06***
Age	-0.01238	0.01861	0.5061
Site	0.04009	0.0901	0.6565
SES	9.02E-05	0.003048	0.9764
Motor Control	0.1933	0.03692	2.12E-07***
WISC-FSIQ	0.04095	0.003566	2.59E-28***

Table S3: PC1 regressed on dimensional measures, with FSIQ included as a covariate

F statistic = 44.57, df = 782, adjusted R^2 = 0.38

variable	b value	SE	p value
(Intercept)	-4.38	0.3911	4.18E-27***
SWAN-IN	-0.09857	0.04616	0.03302*
SWAN-HY	0.01423	0.04851	0.7693
ASSQ	-0.02532	0.007635	0.0009552***
SRS	0.001578	0.002678	0.5558
SCQ	0.01771	0.01181	0.134
Sex	0.4312	0.08984	1.90E-06***
Age	-0.01977	0.01831	0.2806
SES	-0.000572	0.003119	0.8545
Site	0.01253	0.08913	0.8882
Motor Control	0.1836	0.0367	6.99E-07***
WISC-FSIQ	0.04717	0.003191	8.60E-44***

Table S4: PC1 regressed on categorical diagnostic labels with SWAN-inattention and FSIQ included as covariates

F statistic = 36.93, df = 782, adjusted R^2 = 0.39

variable	b value	SE	p value
(Intercept)	-3.582	0.4323	5.14E-16***
ADHD-I	-0.4059	0.1132	0.000356***
ADHD-C	-0.2473	0.1213	0.04179*
ASD	-0.1318	0.1236	0.2867
SLD-Math	-0.3821	0.1107	0.0005829***
SLD-Reading	-0.07789	0.1107	0.4819
Anxiety	-0.01738	0.09335	0.8523
Depression	0.07721	0.159	0.6274
Sex	0.3999	0.09021	1.06E-05***
Age	-0.01249	0.01867	0.5035
Site	0.04107	0.0902	0.649
SES	0.0004573	0.003067	0.8815
Motor Control	0.1932	0.03695	2.20E-07***
SWAN-IN	-0.02529	0.04252	0.5522
WISC-FSIQ	0.04063	0.003605	2.08E-27***

Table S5: Correlations among processing speed tasks, potential confounds and dimensional symptoms

Variable	WISC-Coding	WISC-SS Clinician	WISC-SS EEG	NIH PC
Age	r(1458) = -0.126, ***	r(1450) = -0.086, **	r(841) = -0.127, ***	r(841) = 0.159, ***
Sex	r(1458) = 0.160, ***	r(1450) = 0.068, *	r(841) = 0.095, **	r(841) = 0.074, *
SES	r(1331) = 0.147, ***	r(1319) = 0.151, ***	r(836) = 0.152, ***	r(836) = 0.070
Motor Control	r(1450) = 0.324, ***	r(1437) = 0.321, ***	r(837) = 0.293, ***	r(837) = 0.201, ***
FSIQ	r(1474) = 0.583, ***	r(1462) = 0.536, ***	r(844) = 0.543, ***	r(844) = 0.251, ***
SWAN-IN	r(1341) = -0.172, ***	r(1329) = -0.162, ***	r(840) = -0.200, ***	r(840) = -0.090, *
SWAN-HY	r(1341) = -0.105, ***	r(1329) =-0.088, **	r(840) = -0.133, ***	r(840) = -0.045
ASSQ	r(1342) = -0.139, ***	r(1330) = -0.142, ***	r(840) = -0.182, ***	r(840) = -0.069
SRS	r(1355) = -0.150, ***	r(1343) = -0.165,***	r(842) = -0.189, ***	r(842) = -0.072
SCQ	r(1353) = -0.137, ***	r(1341) = -0.143, ***	r(842) = -0.176, ***	r(842) = 0.002

*all results corrected for multiple comparisons using false discovery rate

Note: though only these five scales are included above, in keeping with consistency with the main paper, values were also examined for the rest of the dimensional symptoms (MFQ, SCARED, ARI). There were no additional associations, apart from SCARED-Parent and ARI-Self being correlated with WISC-SS EEG, both at the * level.

Table S6: Each of the four processing speed tasks regressed separately on categorical diagnostic labels

Without FSIQ:

variable	**WISC-Coding**	**WISC-SS Clinician**	**WISC-SS EEG**	**NIH PC**
(Intercept)	9.34E-151***	4.27E-141***	2.79E-155***	6.54E-47***
ADHD-I	2.78E-07***	0.0053**	0.0001***	0.007**
ADHD-C	0.034*	0.12	0.027*	0.045*
ASD	0.06	0.23	0.23	0.20
SLD-Math	1.11E-13***	2.40E-08***	4.17E-11***	0.13
SLD-Reading	0.00013***	0.0069**	0.0014**	0.23
Anxiety	0.569	0.536	0.51	0.91
Depression	0.47	0.70	0.66	0.34
Sex	4.70E-12***	0.002**	0.30	0.44
Age	0.06	0.25	0.0097**	1.25E-05***
Site	0.94	0.036*	0.00017***	0.42
SES	0.047*	0.0014**	0.1315	0.34
Motor Control	1.13E-10***	1.05E-14***	4.75E-11***	8.52E-08***
F statistic	26.16	17.18	16.89	6.75
df	786	786	786	786
Adjusted R^2	0.27	0.20	0.19	0.08

With FSIQ:

variable	**WISC-Coding**	**WISC-SS Clinician**	**WISC-SS EEG**	**NIH PC**
(Intercept)	3.17E-41***	4.27E-42***	4.47E-44***	5.51E-11***
ADHD-I	5.36E-06***	0.029*	0.0021**	0.019*
ADHD-C	0.051	0.17	0.041*	0.057
ASD	0.061	0.25	0.25	0.18
SLD-Math	0.00017***	0.010*	0.003**	0.95
SLD-Reading	0.28	0.62	0.63	0.97
Anxiety	0.83	0.34	0.76	0.97
Depression	0.75	0.96	0.97	0.43
Sex	3.48E-13***	0.0015**	0.29	0.45
Age	0.057	0.26	0.0078**	8.93E-06***

Site	0.081	0.48	0.033*	0.95
SES	0.67	0.19	0.38	0.96
Motor Control	0.004**	1.55E-06***	0.0023**	0.0003***
WISC-FSIQ	4.23E-22***	2.52E-14***	1.33E-21***	6.91E-05***
F statistic	34.80	21.70	24.94	7.58
df	785	785	785	785
Adjusted R^2	0.36	0.25	0.28	0.10

Table S7: Each of the four processing speed tasks regressed separately on dimensional measures

Without FSIQ:

variable	**WISC-Coding**	**WISC-SS Clinician**	**WISC-SS EEG**	**NIH PC**
(Intercept)	9.54E-129***	9.57E-128***	8.18E-141***	2.61E-39***
SWAN-IN	0.0002***	0.0033**	3.93E-05***	0.095
SWAN-HY	0.65	0.86	0.98	0.40
ASSQ	0.061	0.018*	0.14	0.50
SRS	0.61	0.21	0.54	0.71
SCQ	0.99	0.53	0.84	0.086
Sex	4.11E-12***	0.0064**	0.36	0.19
Age	0.024*	0.17	0.004**	1.98E-06***
SES	0.006**	0.00018***	0.044*	0.16
Site	0.79	0.08	0.0005***	0.44
Motor Control	1.90E-11***	6.67E-16***	3.80E-12***	1.05E-08***
F statistic	18.64	15.43	13.20	7.30
df	783	783	783	783
Adjusted R^2	0.18	0.15	0.13	0.07

With FSIQ:

variable	**WISC-Coding**	**WISC-SS Clinician**	**WISC-SS EEG**	**NIH PC**
(Intercept)	4.98E-36***	2.67E-42***	1.31E-45***	2.20E-10***
SWAN-IN	0.10	0.21	0.03*	0.42
SWAN-HY	0.78	0.98	0.86	0.44
ASSQ	0.006**	0.0024**	0.025*	0.35
SRS	0.74	0.25	0.66	0.64
SCQ	0.24	0.83	0.39	0.032*
Sex	1.44E-14***	0.003**	0.30	0.18
Age	0.012*	0.14	0.0016**	1.49E-06***
SES	0.51	0.26	0.23	0.91
Site	0.031*	0.68	0.042*	0.91
Motor Control	0.016*	3.10E-06***	0.0035**	0.0001***
WISC-FSIQ	5.02E-37***	1.05E-22***	3.78E-32***	2.45E-06***
F statistic	37.15	25.15	28.20	8.87

df	782	782	782	782
Adjusted R^2	0.34	0.26	0.28	0.11

Table S8: Each of the four processing speed tasks regressed separately on categorical diagnostic labels, including SWAN-inattention as a covariate

Without FSIQ:

variable	**WISC-Coding**	**WISC-SS Clinician**	**WISC-SS EEG**	**NIH PC**
(Intercept)	1.32E-149***	7.03E-140***	2.69E-155***	4.49E-46***
ADHD-I	6.39E-05***	0.08	0.040*	0.022*
ADHD-C	0.18	0.48	0.44	0.083
ASD	0.079	0.29	0.33	0.20
SLD-Math	4.63E-13***	5.05E-08***	3.14E-10***	0.13
SLD-Reading	0.0002***	0.0099**	0.0021**	0.25
Anxiety	0.57	0.54	0.57	0.87
Depression	0.40	0.58	0.47	0.33
Sex	9.13E-12***	0.0033**	0.41	0.46
Age	0.053	0.23	0.0076**	1.45E-05***
Site	0.98	0.04*	0.0002***	0.43
SES	0.056	0.0013**	0.17	0.30
Motor Control	1.93E-10***	2.03E-14***	9.26E-11***	1.04E-07***
SWAN-IN	0.20	0.13	0.0061**	0.89
F statistic	24.17	16.12	16.25	6.23
df	783	783	783	783
Adjusted R^2	0.27	0.20	0.20	0.08

With FSIQ:

variable	**WISC-Coding**	**WISC-SS Clinician**	**WISC-SS EEG**	**NIH PC**
(Intercept)	4.88E-40***	4.06E-41***	4.09E-44***	2.04E-10***
ADHD-I	4.59E-05***	0.094	0.044*	0.02*
ADHD-C	0.077	0.32	0.26	0.060
ASD	0.062	0.27	0.30	0.19
SLD-Math	0.0002***	0.009**	0.0037**	0.99
SLD-Reading	0.29	0.67	0.65	0.99
Anxiety	0.82	0.36	0.80	0.99
Depression	0.75	0.89	0.83	0.45
Sex	4.63E-13***	0.0022**	0.37	0.45
Age	0.057	0.26	0.007**	9.12E-06***

Site	0.080	0.47	0.030*	0.96
SES	0.70	0.15	0.40	0.96
Motor Control	0.004**	1.62E-06***	0.0020**	0.0003***
SWAN-IN	0.96	0.62	0.12	0.67
WISC-FSIQ	1.10E-21***	7.63E-14***	2.51E-20***	6.11E-05***
F statistic	32.14	20.20	23.25	7.06
df	782	782	782	782
Adjusted R^2	0.35	0.25	0.28	0.10

Table S9: Correlations between PC2 and four individual PS measures, potential confounds, and dimensional symptoms

**Variable**	**R value**	**P value**	**df**
WISC-Coding	-0.123	0.0003***	844
WISC-SS Clinician	-0.153	7.61E-06***	844
WISC-SS EEG	-0.312	1.30E-20***	844
NIH PC	0.792	8.44E-183***	844
Age	0.248	1.04E-12***	841
Sex	-0.037	0.364	841
SES	0.152	0.224	836
Motor Control	-0.031	0.460	837
FSIQ	-0.147	1.67E-05***	844
SWAN-IN	0.048	0.226	840
SWAN-HY	0.045	0.259	840
ASSQ	0.072	0.053	840
SRS	0.068	0.069	842
SCQ	0.136	0.0001***	842

*all results corrected for multiple comparisons using false discovery rate

Table S10: PC2 regressed on categorical diagnostic labels

Without FSIQ: F statistic = 5.98, DF = 786, adjusted R^2 = 0.07

variable	b value	SE	p value
(Intercept)	-0.9278	0.1942	2.11E-06***
ADHD-I	-0.01212	0.07297	0.8681
ADHD-C	-0.05369	0.07909	0.4975
ASD	0.2002	0.08929	0.02525*
SLD-Reading	0.2217	0.07425	0.002923**
SLD-Math	0.07385	0.0768	0.3365
Anxiety	-0.005792	0.06743	0.9316
Depression	0.07433	0.1145	0.5163
Sex	-0.07299	0.06517	0.2631
Age	0.08039	0.01348	3.75E-09***
SES	-0.06147	0.06418	0.3385
Site	-0.0009114	0.00214	0.6703
Motor Control	0.01964	0.02497	0.4317

With FSIQ: F statistic = 5.84, df = 785, adjusted R^2 = 0.07

variable	b value	SE	p value
(Intercept)	-0.4494	0.3085	0.1455
ADHD-I	-0.02518	0.07312	0.7306
ADHD-C	-0.05847	0.07898	0.4594
ASD	0.1983	0.08912	0.02635*
SLD-Reading	0.163	0.07974	0.04124*
SLD-Math	0.02869	0.07994	0.7198
Anxiety	-0.0005098	0.06736	0.994
Depression	0.08449	0.1144	0.4603
Sex	-0.07237	0.06505	0.2663
Age	0.08018	0.01346	3.86E-09***
SES	-0.03767	0.06517	0.5634
Site	0.0001767	0.002204	0.9361
Motor Control	0.03876	0.0267	0.147
WISC-FSIQ	-0.005142	0.002579	0.04654*

Table S11: PC2 regressed on dimensional measures

Without FSIQ: F statistic = 6.22, df = 783, adjusted R^2 = 0.06

variable	b value	SE	p value
(Intercept)	-1.029	0.198	2.58E-07***
SWAN-IN	0.03045	0.0329	0.3549
SWAN-HY	0.02651	0.03515	0.4509
SRS	0.003108	0.005525	0.5739
SCQ	-0.00179	0.00194	0.3566
ASSQ	0.01676	0.008523	0.04957*
Sex	-0.03367	0.0651	0.6052
Age	0.08787	0.01327	6.60E-11***
SES	-0.0008499	0.002177	0.6964
Site	-0.05128	0.06395	0.4229
Motor Control	0.01984	0.02493	0.4265

With FSIQ: F statistic = 6.51, df = 782, adjusted R^2 = 0.07

variable	b value	SE	p value
(Intercept)	-0.4303	0.282	0.1275
SWAN-IN	0.01258	0.03328	0.7057
SWAN-HY	0.02822	0.03498	0.4201
SRS	0.00397	0.005505	0.471
SCQ	-0.001686	0.001931	0.3828
ASSQ	0.01456	0.008513	0.08756
Sex	-0.0342	0.06478	0.5977
Age	0.08793	0.0132	5.20E-11***
SES	0.0009451	0.002249	0.6745
Site	-0.02454	0.06427	0.7027
Motor Control	0.04717	0.02646	0.07505
WISC-FSIQ	-0.00683	0.002301	0.003087**
